# Substance use disorders in Arab countries: research activity and bibliometric analysis

**DOI:** 10.1186/1747-597X-9-33

**Published:** 2014-08-23

**Authors:** Waleed M Sweileh, Sa’ed H Zyoud, Samah W Al-Jabi, Ansam F Sawalha

**Affiliations:** 1 Department of Pharmacology and Toxicology, College of Medicine and Health Sciences, An-Najah National University, Nablus, Palestine; 2 Department of Clinical and Community Pharmacy, College of Medicine and Health Sciences, An-Najah National University, Nablus, Palestine

**Keywords:** Bibliometric, Substance use disorders, Arab countries, ISI web of science

## Abstract

**Background:**

Substance use disorders, which include substance abuse and substance dependence, are present in all regions of the world including Middle Eastern Arab countries. Bibliometric analysis is an increasingly used tool for research assessment. The main objective of this study was to assess research productivity in the field of substance use disorders in Arab countries using bibliometric indicators.

**Methodology:**

Original or review research articles authored or co-authored by investigators from Arab countries about substance use disorders during the period 1900 – 2013 were retrieved using the ISI Web of Science database. Research activity was assessed by analyzing the annual research productivity, contribution of each Arab country, names of journals, citations, and types of abused substances.

**Results:**

Four hundred and thirteen documents in substance use disorders were retrieved. Annual research productivity was low but showed a significant increase in the last few years. In terms of quantity, Kingdom of Saudi Arabia (83 documents) ranked first in research about substance use disorders while Lebanon (17.4 documents per million) ranked first in terms of number of documents published per million inhabitants. Retrieved documents were found in different journal titles and categories, mostly in *Drug and Alcohol Dependence* Journal. Authors from USA appeared in 117 documents published by investigators from Arab countries. Citation analysis of retrieved documents showed that the average citation per document was 10.76 and the *h -* index was 35. The majority of retrieved documents were about tobacco and smoking (175 documents) field while alcohol consumption and abuse research was the least with 69 documents.

**Conclusion:**

The results obtained suggest that research in this field was largely neglected in the past. However, recent research interest was observed. Research output on tobacco and smoking was relatively high compared to other substances of abuse like illicit drugs and medicinal agents. Governmental funding for academics and mental health graduate programs to do research in the field of substance use disorders is highly recommended.

## Background

The Diagnostic and Statistical Manual of Mental Disorders V (DSM V) published in 2013 has eliminated the separate categories of substance abuse and substance dependence and replaced them with one unified category called substance use disorders [1]. The term substance use disorders refer to the use of one or more substances leading to a clinically significant impairment or distress [1]. The DSM-V recognizes ten separate classes of drugs that can lead to substance use disorders. These classes include: alcohol, caffeine, cannabis, hallucinogens, inhalants, opioids, sedatives, hypnotics, anxiolytics, stimulants, and other or unknown substances. Substance-related disorders are not limited to any particular country or world region. For example, in the Eastern Mediterranean Region (EMR), drug use disorders is common and accounting for a loss of 4 disability-adjusted life years (DALYs) and 9 deaths per 1000 population, compared with the loss of 2 DALYs and 4 deaths per 1000 population globally [2]. The EMR includes people with different races, ethnicities, religions and cultures with different languages and habits. Of particular interest in the EMR is Arab region which extend from the Atlantic Ocean in the West to the Arabian Sea in the East, and from the Mediterranean Sea in the North to the Horn of Africa and the Indian Ocean in the Southeast [3]. Arabs share the same language, culture, religion and historical background. Therefore, they are frequently considered as one unit despite the differences in wealth and population size [3]. Over the last few decades, medical education and medical services have witnessed great positive change in Arab region, particularly in Arab countries with politically and economically stable conditions [4-6]. This positive change should be reflected on various medical research activities including those pertaining to substance-related disorders. Arab countries, represented by Arab league, are collaborating with the United Nations office on Drugs and Crime (UNODC) to combat illicit drugs and human trafficking. In fact, on December 2010, the UNODC and the Secretary General of the League of Arab States officially launched a 100 million USD five-year Regional program on Drug Control, Crime Prevention and Criminal Justice Reform for the Arab States for the period 2011–2015 directed mainly toward countering illicit trafficking, organized crime and terrorism; promoting integrity and building justice; and drug prevention and health [7]. This collaboration and funding is important to promote research in the field of substance use disorders. Actually, quality and quantity of research output in substance use disorders reflect country’s interest and efforts to provide better services and health standards to the people of that country. A commonly used method to assess research output from any country or region is bibliometric analysis which refers to the implementation of statistical tools to evaluate research productivity [8,9]. Bibliometric analysis has been applied to various medical topics and is now widely accepted as a method of measuring research and literacy output in any particular research area [10-13]. Based on our knowledge, no bibliometric studies in the field of substance-related disorders have been carried out in Arab countries. Such studies are important because they will lead to better understanding of the current and future status of substance-related disorders in Arab region. Furthermore, the results of such studies will help health policy makers to draw plans to combat substance-related disorders. In addition, the momentum of research activity needs to be maintained through continuous analysis of publications from researchers. Therefore, we carried out this study to assess substance-related disorders research activity in Arab countries using bibliometric indicators to shed light on the status of research in this field and to enlighten investigators and policy makers in this regard.

## Methodology

The data used in this study were based on the ISI Web of Science (WoS), which is one of the world largest databases of peer-reviewed literature [14]. The search strategy employed was based on using advanced search tool in WoS and using specific codes to retrieve the required scientific documents. The WoS allows researchers to use codes such as WC (WoS research category) or TI (title search) or TS (topic search) in advanced search strategy. Furthermore, WoS allows researchers to refine results by exclusion or limiting analysis to certain particular documents. Web of Science is easy to use and allows researchers to do analysis regarding annual research productivity, source titles in which documents were published, countries which published the retrieved documents, the languages of the documents, total citation for the retrieved documents, the average citation per document and the h-index of the retrieved documents. It is important here to note that the choice to use WoS to retrieve research documents about substance abuse instead of PubMed or Scopus was based on several reasons. First, WoS allows researchers to retrieve documents based on research category which is not doable with either PubMed or Scopus. For example, WoS has a search category called “substance abuse” which encompasses all journals in the field of substance abuse. Similarly, WoS has other research categories like “psychiatry”, “chemistry” and others which allow researchers to refine results in an accurate way. Second, WoS is a rich database that includes leading and high impact journals in scientific field [15-19]. Journals indexed in ISI WoS are considered internationally leading and powerful journals with international reputation and impact in the field of substance abuse. Third, in contrast to PubMed, WoS covers most scientific publication and not only the medical and biomedical publication. Finally, WoS covers the oldest publications and its records go back to 1900. A comprehensive analysis of the advantages and disadvantages of various databases including WoS, PubMed, and Scopus is presented by Falagas et al. [16].

The strategy followed in this project to retrieve published documents about substance abuse originating from Arab countries consisted of seven steps shown in Additional file 1. In this project, and to avoid false positive results, documents retrieved were limited to include original research articles and review articles only while editorials, conference papers, book chapters were excluded because some of them might have been published as original articles. Furthermore, all Arab countries were entered in the advanced search tool except for Palestine which is not recognized by ISI WoS as an independent country and is not shown in the list of countries available for search in ISI WoS. At the start of the search strategy, we set the date of the search to be from 1900 – 2013.

Four steps were carried out to retrieve published documents about substance use disorders authored or co-authored by investigators from Arab countries (Additional file 1). Using this strategy, documents published in substance abuse journal category as well as documents published in other journal categories will be retrieved as well. This complex approach was needed because substance use disorders is a multidisciplinary field and cannot be retrieved from a set of core journals grouped into the WoS category called “substance abuse”. Actually, many documents in substance use disorders are published in journals of psychiatry, psychology, public health or behavioral science journals. Therefore, a multi step approach is needed to retrieve published documents with high validity. Such approach is challenging because some of the key words like benzodiazepines or alcohol might be encountered in other disciplines like chemistry. Fortunately, the WoS has the power to refine and exclude documents pertaining to any irrelevant discipline like chemistry or physics for example. The steps used to retrieve substance use disorders research documents were as follows:

1. In the first step, a WoS search category was performed. The WoS has a search category called “substance abuse”. This category includes all journals in the field of substance abuse. Therefore, in the first step we searched for documents from Arab countries that were published in substance abuse journal category. The search is limited to original and review articles. This step is shown as step 1 in Additional file 1 and looks like this: *((CU = (Jordan) OR CU = (Iraq) OR CU = (Syria) OR CU = (Saudi) OR CU = (Kuwait) OR CU = (Egypt) OR CU = (Yemen) OR CU = (Qatar) OR CU = (Emirates) OR CU = (Bahrain) OR CU = (Oman) OR CU = (Sudan) OR CU = (Tunisia) OR CU = (Algeria) OR CU = (Lebanon) OR CU = (Libya) OR CU = (Morocco) OR CU = (Somalia) OR CU = (Djibouti) OR CU = (Comoros) OR CU = (Mauritania)) AND WC = (substance abuse)) AND DOCUMENT TYPES* ***:*** *(Article OR Review)*. The code CU refers to country name.

2. In the second step, a title search was performed. The selected words used for title search in WoS were those pertaining to substance abuse. The second step is shown in Additional file 1 and looks like this: ((CU = (Jordan) OR CU = (Iraq) OR CU = (Syria) OR CU = (Saudi) OR CU = (Kuwait) OR CU = (Egypt) OR CU = (Yemen) OR CU = (Qatar) OR CU = (Emirates) OR CU = (Bahrain) OR CU = (Oman) OR CU = (Sudan) OR CU = (Tunisia) OR CU = (Algeria) OR CU = (Lebanon) OR CU = (Libya) OR CU = (Morocco) OR CU = (Somalia) OR CU = (Djibouti) OR CU = (Comoros) OR CU = (Mauritania)) AND TI = (“substance abuse” OR “substance use” OR “drug abuse” OR “abstinen*” OR addict* OR “drug use” OR “drug dependence” OR “illicit drugs” OR “street drugs”)) AND DOCUMENT TYPES: (Article OR Review)

3. In the third step, a title search was performed using words related to substances or drugs commonly encountered in substance abuse. Since these words belong to different scientific disciplines, the results obtained were limited to journal categories belonging to public heath, substance abuse, psychiatry, behavioral sciences and psychology. At the same time, documents belonging to chemistry, engineering, technology, agriculture, water resources, and physics journal categories were excluded. The third step looks like this: ((CU = (Jordan) OR CU = (Iraq) OR CU = (Syria) OR CU = (Saudi) OR CU = (Kuwait) OR CU = (Egypt) OR CU = (Yemen) OR CU = (Qatar) OR CU = (Emirates) OR CU = (Bahrain) OR CU = (Oman) OR CU = (Sudan) OR CU = (Tunisia) OR CU = (Algeria) OR CU = (Lebanon) OR CU = (Libya) OR CU = (Morocco) OR CU = (Somalia) OR CU = (Djibouti) OR CU = (Comoros) OR CU = (Mauritania)) AND TI = ((alcoh*) OR (tobac*) OR (smok*) OR (snuff) OR (cigarette) OR (smoker) OR (nicotine) OR (hookah) OR (narghile) OR (argila) OR (shisha) OR (waterpipe) OR (marijuana) OR (THC) OR (ecstasy) OR (MDMA) OR (LSD) OR (PCP) OR (amphet*) OR (cocaine) OR (opioid*) OR (opiate) OR (narco*) OR (khat) OR (Qat) OR (crack) OR (heroin) OR (barbit*) OR (mescaline) OR (benzodiazepines) OR (diazepam) OR (codeine) OR (methylphenidate) OR (psychoactive*) OR (cannabis) OR (inhalants) OR (hallucinogens) OR (cannabinol) OR (hashish) OR (caffeine) OR (sedatives) OR (hypnotics) OR (anxiolytics) OR (tramadol))) AND ***DOCUMENT TYPES:*** *(Article OR Review)* ***Refined by: WEB OF SCIENCE CATEGORIES:*** *(BEHAVIORAL SCIENCES OR PUBLIC ENVIRONMENTAL OCCUPATIONAL HEALTH OR SUBSTANCE ABUSE OR PSYCHIATRY OR PSYCHOLOGY CLINICAL OR PSYCHOLOGY MULTIDISCIPLINARY OR PSYCHOLOGY) AND [excluding]* ***WEB OF SCIENCE CATEGORIES:*** *(ENGINEERING ENVIRONMENTAL OR ERGONOMICS OR ENGINEERING INDUSTRIAL OR ENVIRONMENTAL SCIENCES OR CONSTRUCTION BUILDING TECHNOLOGY OR WATER RESOURCES OR AGRICULTURE DAIRY ANIMAL SCIENCE)*

4. In the fourth step, results in step 1, 2 and 3 were combined and the results were analyzed and presented. The total number of documents retrieved was 413 **(**See Additional file 1).

For simplification process, we divided substances of abuse into four groups: (1) alcohol group; (2) illicit and medicinal drugs group; (3) tobacco group and (4) miscellaneous research documents that do not fit in any of the previous three groups. We searched the results obtained in step number 4 for documents that belong to each group and were analyzed and presented. The search for documents belonging to each group was shown in Additional file 1 in which we used the title search (TI) to search for documents belonging to each group within the results obtained in step # 4.

Once the required results were obtained, the data were transferred into Microsoft Excel for further analysis and presentation. In this project, we presented results as rank order using the standard competition ranking (SCR) in which only the 10 top ranked was taken into consideration. The *h*-index for data was also presented. The *h*-index is a country’s number of articles (*h*) that have received at least *h* citations [20]. Publication activity was adjusted for Arab countries based on population size and therefore the total number of published documents per million inhabitants was presented. The number of population for each country was obtained from the online databases of the World Bank [21]. Regarding the ethical issues, the Institutional review Board (IRB) requested no file submission for such a study since it imposes no risk on human subjects. Finally, the indices used in this study to assess the quantity and quality of worldwide research output about green tea were (1) annual publication pattern; (2) journals in which substance abuse research was published; (3) contribution of each Arab country to substance abuse research; and finally (4) citation analysis of substance abuse research originating from countries.

## Results

**Figure 1:**
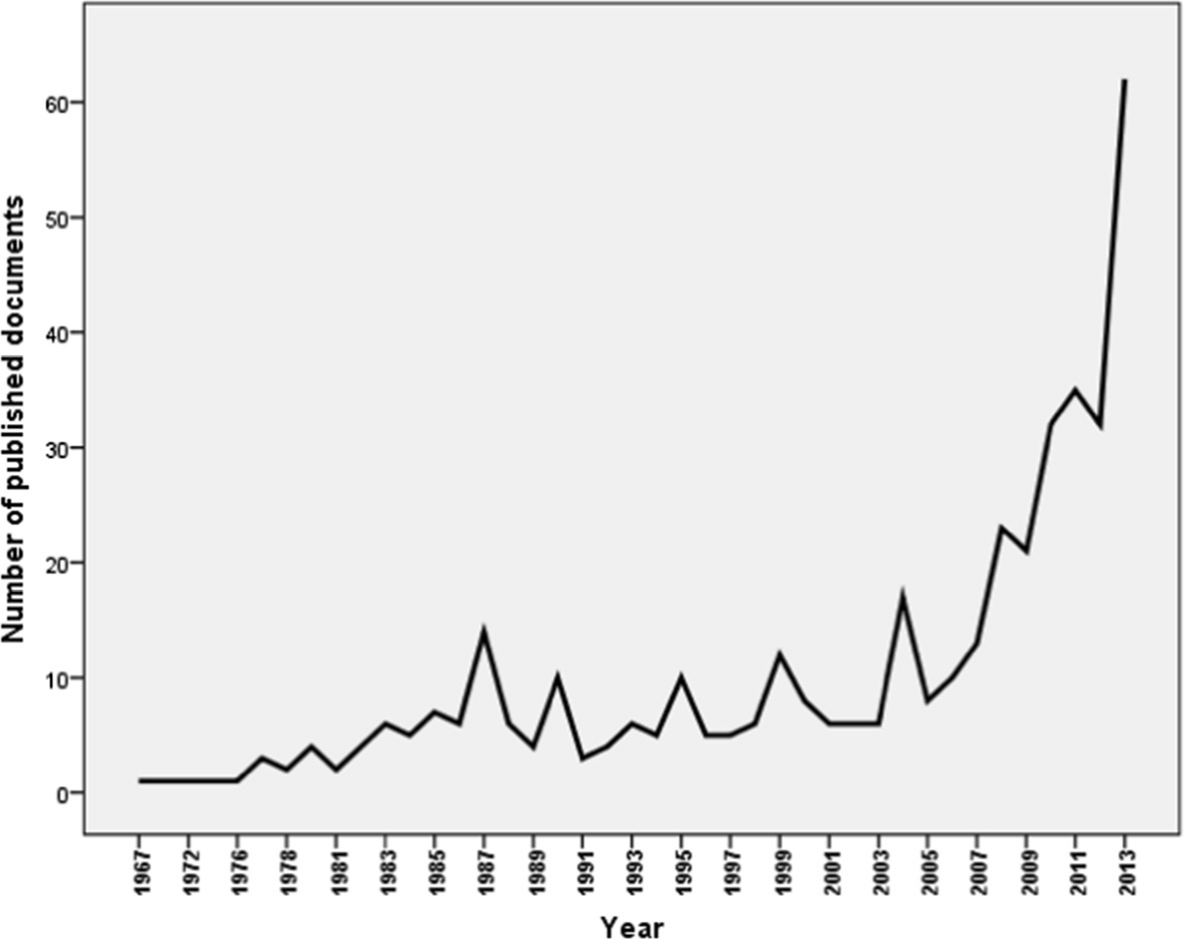
Annual growth of substance use disorders research in Arab countries.

The total number of documents retrieved from Arab countries using the methodology stated and was 413. The results consisted of 401 original research articles and 12 review articles. The majority of retrieved documented were written in English (398; 96.37%). A small number of documents (15; 3.63%) were written in French language. Of the 413 retrieved documents, 40 (9.68%) were available as open access while the remaining were not. Research about substance-related disorders in Arab countries started as early as 1967. The annual number of published documents remained low and fluctuating but showed a significant rise in the last few years. The maximum annual number of published documents was recorded in year 2013 with 62 documents. Table 1 showed the total number of documents published from Arab countries in substance-related disorders presented in different time intervals while Figure 1 showed the annual growth in the field of substance use disorders in Arab countries.

**Table 1:** Number of published documents with time

**Period**	**Number of published documents (%)**
	**N = 413**
≤ 1980	14 (3.38)
1981 - 1990	64 (15.49)
1991 - 2000	64 (15.49)
2001 - 2010	142 (45.22)
2011 - 2013	129 (31.23)

Contribution of each Arab country to substance abuse research along with *h*-index for publications from each country is shown in Table 2. Kingdom of Saudi Arabia with a total of 83 published documents ranked number one in terms of quantity of publications followed by Lebanon and Egypt. Three Arab countries, Mauritania, Djibouti and Comoros had no contribution to the field. When data was stratified based on total population and represented as number of documents per million inhabitants, Lebanon ranked first followed by Jordan and Qatar. Lebanon also ranked first in quality of substance use research as measured by *h*-index. The most productive institution in substance-related disorders research in Arab countries was American University of Beirut (52 documents) followed by King Saud University (34 documents). Collaboration between USA researchers and researchers from Arab countries was apparent. Authors from USA appeared in 117 documents published by investigators from Arab countries. Other collaborating countries were France (21 documents), Canada (21 documents) and Germany (20 documents). Citation analysis showed that the sum of citations of the 413 documents published from the Arab countries was 4444 including self citation. The average citation per document was 10.76. The *h* index of the 413 documents was 35 at the time of data analysis (Table 3).

**Table 2:** Contribution of each Arab country to substance abuse research measured by number of documents published and number of documents published per million

**Arab country**	**Number of population in millions**	**Number of published documents**	**Number of published documents per million**	***h *** **index**
Kingdom of Saudi Arabia	28.29	83	2.93	11
Lebanon	4.43	77	17.4	21
Egypt	80.72	71	0.88	12
Kuwait	3.25	39	12	10
Syria	22.4	35	1.56	17
United Arab Emirates	9.21	35	3.8	10
Jordan	6.32	31	4.91	6
Tunisia	10.78	12	1.11	3
Morocco	32.52	12	0.37	4
Qatar	2.05	9	4.39	3
Iraq	32.58	8	0.25	2
Sudan	37.2	8	0.22	4
Yemen	23.85	7	0.29	3
Algeria	38.48	6	0.16	4
Bahrain	1.32	5	3.79	2
Somalia	10.2	4	0.39	3
Oman	3.31	3	0.91	2
Libya	6.16	1	0.16	1

**Table 3:** Citation analysis of retrieved documents

**Variable**	**Result**
Number of document found	413
Sum of the times cited	4444
Sum of times cited without self-citations	3748
Citing articles	2662
Citing articles without self-citations	2477
Average citations per item	10.76
h-index	35

**Figure 2:**
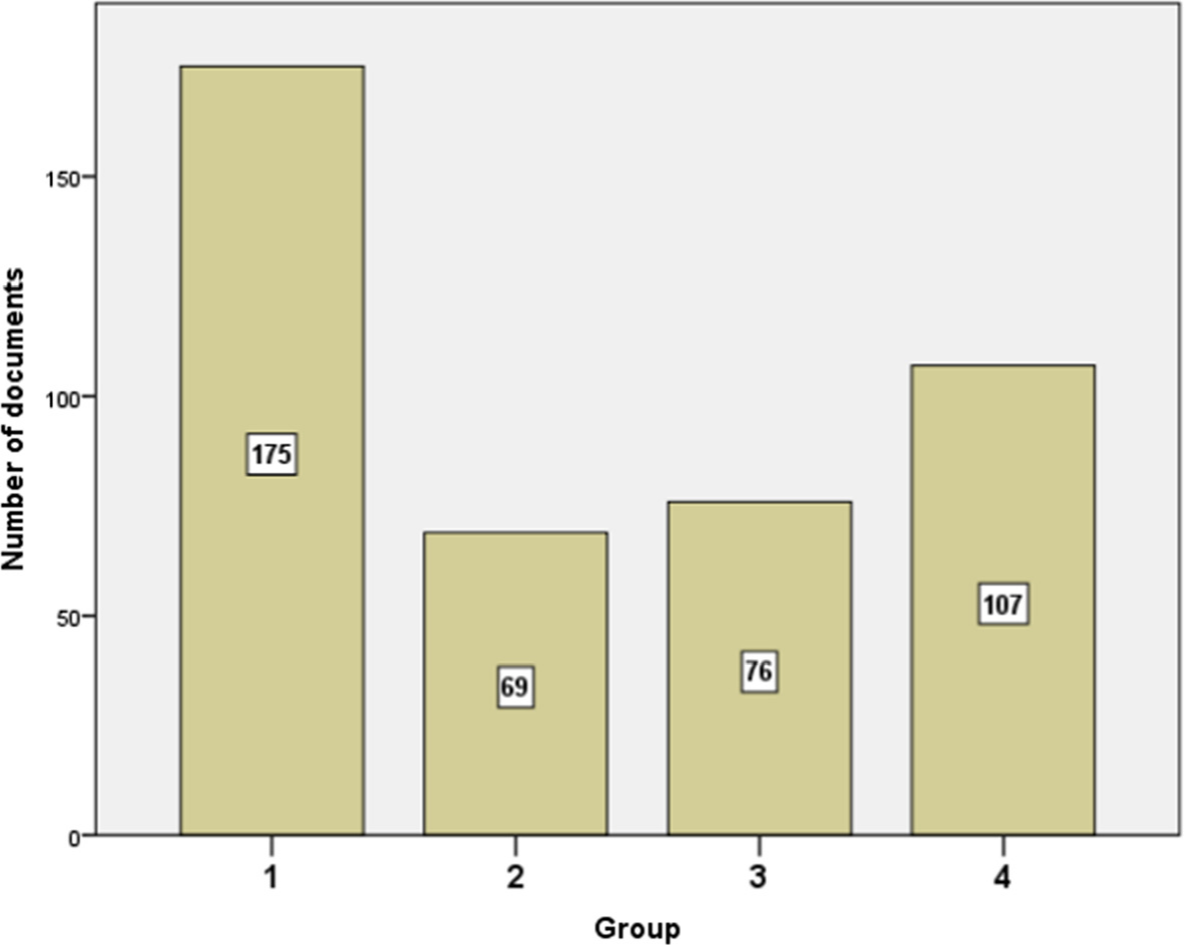
**Number of published documents in substance-related disorders stratified by topic.** Group 1: Tobacco or Waterpipe research documents; Group 2: Alcohol research documents; Group 3: illicit drugs and abused medicinal agents research documents; Group 4: miscellaneous research topics. Note: total results exceed 413 because of some overlap in results of the first three groups.

Top 10 journals in which the retrieved documents were published are shown in Table 4. Journal of *Drug and Alcohol Dependence* ranked first followed by *Nicotine and Tobacco Research* journal. The fact that *BMC Public Health* journal ranked 4th in the top 10 list indicated that the strategy of retrieval of published substance abuse documents from Arab countries succeeded in retrieving documents published outside substance abuse journal category. Of particular importance is the appearance of 2 journals in the field of tobacco in the top 10 journals suggesting high volume of tobacco research publications from Arab countries. Of the 413 retrieved documents, 175 documents were in the search topic of tobacco or waterpipe (group 1), 69 documents were about alcohol (group 2), and 76 documents were about illicit or medicinal drug abuse (group 3). Of course, there was some overlap between results obtained for groups 1, 2 and 3. The remaining 107 documents were about substance abuse and addiction in general, factors associated with substance abuse and epidemiology of substance abuse, or complications of substance abuse (Figure 2). Low research productivity about alcohol abuse is expected since alcoholism is not an accepted social behavior in Arab countries due to religious reasons.

**Table 4:** Top 10 journals in which substance abuse documents were authored or co-authored by an investigator from Arab countries

**SCR**^**a**^	**Journal**	**Number of documents (%)**
		**N = 413**
**1st**	*Drug and Alcohol Dependence*	36 (8.72)
**2nd**	*Nicotine Tobacco Research*	23 (5.57)
**3rd**	*Research Communications in Substances of Abuse*	17 (4.12)
**4th**	*Tobacco Control*	11 (2.66)
**4th**	*BMC Public Health*	11 (2.66)
**6th**	*Pharmacology Biochemistry and Behavior*	9 (2.18)
**6th**	*Addictive Behaviors*	9 (2.18)
**8th**	*Preventive Medicine*	8 (1.94)
**8th**	*Bulletin on Narcotics*	8 (1.94)
**10th**	*Substance Use Misuse*	7 (1.70)

^a^ Equal journals have the same ranking number, and then a gap is left in the ranking numbers.

## Discussion

Arab countries, like other regions in the world, face several future health challenges particularly those pertaining to psychiatry and mental health problems such as substance-related disorders [22-27]. No doubt that political instabilities, social pressure and insecurity are creating an uncomfortable atmosphere that leads to several psychiatric problems in Arab countries [28]. Studies from Arab countries have reported a wide variety of substances and medications being abused. For example, abuse of tramadol in Egypt and other Middle Eastern countries have reached an alarming limit [29]. Another example is substance use disorders in Kingdom of Saudi Arabia which has been considered a public health problem with serious social consequences [30].

Our results show that the annual research productivity in the field of substance-related disorders from the Arab countries has been low for a long time but witnessed a significant increase in the past few years. This might be due to low prevalence of substance use disorders in Arab countries or lack of interest of Arab researchers in substance–related disorders research. Our findings is in agreement with an editorial published recently and indicated that substance abuse research in Arab world is limited [31]. Low research output in the field of substance-related disorders from Arab region might suggest lack of good mental health services and rehabilitation centres for substance abuse and addiction in the Arab countries [22]. A study carried out in Egypt found that patients with substance dependence had a significantly worse overall quality of life than the WHO standards [32].

In Muslim community, spiritual healing and advice from traditional and religious personnel remain a common practice for psychiatric and substance-related problems rather than evidence based therapy for substance-related disorders [33]. In most Arab countries, use of illicit drugs and substance-related disorders are considered a deviation from the recommendation of the holy Quran. However, despite restricting religious and social factors, use of alcohol and illicit drugs has been reported from Arab and Islamic countries [34,35]. Actually, substance use problem is considered a serious public health in Saudi Arabia and that school- and community-based prevention programs are highly required in Saudi Arabia as a first-line strategy in the prevention of substance abuse [30]. The increased awareness about substance use disorders, like tramadol story in Egypt and Gaza, have led to the recent significant increase in research and interest about substance use disorders in Arab countries [29].

Unfortunately, epidemiological studies on substance use disorders are rare from Arab countries which make comparison of research productivity and prevalence rates of substance use disorders difficult to do [36]. However, the WHO ATLAS on substance use disorders provide epidemiologic data regarding alcohol and drug use disorders obtained from available governmental data and available published literature [2]. For example, in KSA, Egypt and Jordan, prevalence estimates of alcohol use disorders (12-month prevalence, %) among females older than 15 years old was zero while among males older than 15 years old was 0.44 in Egypt, 0.38 in KSA and 0.32 in Jordan. On the other hand, prevalence estimates of drug use disorders were 1.3, 0.63, and 0.01 among males in Egypt, Jordan and KSA respectively. Drug use disorders were reported among females in Egypt and Jordan but not in KSA [2]. These prevalence estimates were lesser than that reported from Israel [2].

It was not surprising that countries like Kingdom of Saudi Arabia and Egypt ranked top in quantity of substance use disorders research since these two countries ranked top in several other fields [37,38]. Of course, population size in Egypt and huge economy of KSA contributed to this relatively high research output. The fact that Lebanon ranked first in number of documents per million inhabitants suggests that there is active research groups in substance use disorders in Lebanon. The result that American University in Beirut ranked top institution in substance use disorders in Arab countries endorses the finding that Lebanon has the highest number of substance use documents per million inhabitants. Another potential reason for Lebanon being number one in research output per million inhabitants is the potential collaboration of researchers in Lebanon, particularly those at the American University of Beirut with other investigators in USA which is evident in many of their publications. Authors from Arab countries mainly collaborated with authors from United States of America. This may be because most academics in Arab world graduated from or were trained in these countries. The variations in research output among different Arab countries in the field of substance use disorders is due to several factors including national policies toward research in general and national policies regarding prevention of substance abuse and drug trafficking and border security on the other hand. Prevention of such problems requires identification of the substances of abuse and types of illicit substances being smuggled across borders as well as their sources in the community.

Our results show that most published documents about substance use disorders from Arab countries were in the field of tobacco smoking and waterpipe use. This is expected since a bibliomtric analysis about tobacco in Middle East countries using Scopus based tool has retrieved approximately 500 documents about tobacco research [39]. The discrepancy between the results obtained in the current study and those published by Zyoud et al. is probably due to several reasons including the search engine used and the fact that we were looking at tobacco use within the context of substance use disorders as evident in the methodology rather than tobacco use and smoking in general [39]. This is also evident in top 10 journals of tobacco research in Zyoud et al. article which were mainly in the field of public health or journals that were not indexed in ISI WoS such as *Eastern Mediterranean Health Journal*. This is in contrast to the top 10 journals obtained in the current study which were mainly in the field of substance use disorders which again endorse the methodology that we used in retrieval of substance use disorders from Arab countries.

It was surprising that research about illicit drugs and medicinal agents abuse like morphine, amphetamine, volatile substances, inhalants and others were less than half that of substance use disorders related to tobacco despite that such substances pose real threat to the society aside from their health consequences. Moreover, abuse of illicit drugs such as stimulants, sedatives, hypnotics and others have been reported from Arab countries. A study among Saudi patients in addiction treatment settings indicated that the most commonly abused substances were amphetamine (4–70.7%), heroin (6.6–83.6%), alcohol (9–70.3%) and cannabis (1–60%) [30]. In Egypt, abuse of volatile substances was common and was reported among children who considered that these volatile liquids such as glue to be inexpensive and legal to use [40]. Khat in Yemen and some other African problem is a social, economic and health problem [41,42]. However, research about khat and other related illicit substances and medicinal agents published from Arab countries was relatively lower than that of tobacco and was comparable to that of alcohol as evident in the results obtained in the current study. Research about alcoholism and alcohol abuse was the least type of substance use published documents. However, this does not mean that Arab countries do not have such a problem. No doubt that fear of God and religious values limits alcohol abuse problem in Arab countries [43]. The extent of alcohol use problems in Saudi Arabia was reportedly considerable [44].

Like other bibliometric analysis studies, our current study has few limitations. Although the ISI WoS is a reputable database, it does not include all journals and some publications in the field of substance use disorders might have been missed. In the Arab world, there are several medical journals that are not indexed in ISI WoS and whose publications were not counted. For example, reports about substance abuse published in the *Arab Journal of Psychiatry* or *Eastern Mediterranean Health Journal* were not counted because they are not indexed in ISI WoS. The methodology developed in this article did not distinguish between substance use and substance dependence and we retrieved documents in substance abuse, addiction altogether. We are also limited by the key words used in search methodology. These key words may or may not include or exclude relevant documents. The only method to avoid false positive errors is to do a manual review of all retrieved documents. The authors did a quick manual review of a random sample of retrieved documents to ensure validity and we concluded that there is no reason to believe that there was a selection error or bias. Despite these limitations, our study is the first of its type from an area with volatile situation where data in this field are highly needed to formulate a future policy on drug use, drug prescription, drug control and border security.

## Conclusions

Substance use disorders are one of the future challenging problems to Arab countries and data pertaining to this field are of great importance. The results obtained in the current study suggest that research in this field was largely neglected in the past. However, recent research interest was observed. Research output on tobacco and smoking was relatively high compared to other substances of abuse like illicit drugs and medicinal agents. Arab countries need to invest more research in this field and need to invest in more international collaboration to help make substance use disorders in Arab countries more visible to policy makers and international agencies. Furthermore, health authorities in Arab countries need to observe misuse of prescribed medicinal agents for potential abuse and implement prescription policies to limit potential abuse. Governmental funding for academics and mental health graduate programs to do research in the field of substance use disorders is also needed.

## Abbreviations

ISI: Institute for scientific information; USA: United States of America; KSA: Kingdom of Saudi Arabia; IRB: Institutional review board; UNODC: United Nations office on drugs and crime; NIDA: National Institute of Drug Abuse; NIAAA: National Institute on alcohol abuse and alcoholism; DSM: Diagnostic and statistical manual of mental disorders; WoS: Web of science; SCR: Standard competition ranking.

## Competing interests

The authors declare that they have no competing interests.

## Authors’ contributions

All authors were involved in drafting the article, and all authors approved the final version to be submitted for publication. WS and SA participated in the study design, and provided critical revision of manuscript for important intellectual content. SA and AS were involved in manuscript preparation, writing and critique.

## Supplementary Material

Additional file 1 Methodology used to retrieve documents for analysis using ISI WoS.cmt. Click here for file
