# Supplementary material for: Substance use disorders in Arab countries: research activity and bibliometric analysis
Source: Subst Abuse Treat Prev Policy. 2014 Aug 23;9:33. doi: 10.1186/1747-597X-9-33 (PMC4144697; doi:10.1186/1747-597X-9-33)
Supplement: Additional file 1 — Methodology used to retrieve documents for analysis using ISI WoS.cmt. [file 1747-597X-9-33-S1.doc]

Additional file 1 **Methodology used to retrieve documents for analysis using ISI WoS**

| # 1 | [**179**](http://apps.webofknowledge.com/summary.do?product=WOS&doc=1&qid=10&SID=3D4lpT9QyE9RjUNwMDP&search_mode=AdvancedSearch) | ((CU=(Jordan) OR CU=(Iraq) OR CU=(Syria) OR CU=(Saudi) OR CU=(Kuwait) OR CU=(Egypt) OR CU=(Yemen) OR CU=(Qatar) OR CU=(Emirates) OR CU=(Bahrain) OR CU=(Oman) OR CU=(Sudan) OR CU=(Tunisia) OR CU=(Algeria) OR CU=(Lebanon) OR CU=(Libya) OR CU=(Morocco) OR CU=(Somalia) OR CU=(Djibouti) OR CU=(Comoros) OR CU=(Mauritania)) AND WC=(substance abuse)) *AND***DOCUMENT TYPES:** (Article OR Review)  *Indexes=SCI-EXPANDED, SSCI, A&HCI, CPCI-S, CPCI-SSH, BKCI-S, BKCI-SSH, CCR-EXPANDED, IC Timespan=1900-2013* |
| --- | --- | --- |
| # 2 | [**102**](http://apps.webofknowledge.com/summary.do?product=WOS&doc=1&qid=104&SID=3D4lpT9QyE9RjUNwMDP&search_mode=AdvancedSearch) | ((CU=(Jordan) OR CU=(Iraq) OR CU=(Syria) OR CU=(Saudi) OR CU=(Kuwait) OR CU=(Egypt) OR CU=(Yemen) OR CU=(Qatar) OR CU=(Emirates) OR CU=(Bahrain) OR CU=(Oman) OR CU=(Sudan) OR CU=(Tunisia) OR CU=(Algeria) OR CU=(Lebanon) OR CU=(Libya) OR CU=(Morocco) OR CU=(Somalia) OR CU=(Djibouti) OR CU=(Comoros) OR CU=(Mauritania)) AND TI=("substance abuse" OR "substance use" OR "drug abuse" OR "abstinen*" OR addict* OR "drug use" OR "drug dependence" OR "illicit drugs" OR "street drugs")) AND DOCUMENT TYPES: (Article OR Review)  Indexes=SCI-EXPANDED, SSCI, A&HCI, CPCI-S, CPCI-SSH, BKCI-S, BKCI-SSH, CCR-EXPANDED, IC Timespan=1900-2013 |
| # 3 | [**277**](http://apps.webofknowledge.com/summary.do?product=WOS&doc=1&qid=137&SID=3D4lpT9QyE9RjUNwMDP&search_mode=Refine) | TI= (marijuana) OR (THC) OR (ecstasy) OR (MDMA) OR (LSD) OR (PCP) OR (amphet*) OR (cocaine) OR (opioid*) OR (opiate) OR (narco*) OR (khat) OR (Qat) OR (crack) OR (heroin) OR (barbit*) OR (mescaline) OR (benzodiazepines) OR (diazepam) OR (codeine) OR (methylphenidate) OR (psychoactive*) OR (cannabis) OR (inhalants) OR (hallucinogens) OR (cannabinol) OR (hashish) OR (caffeine) OR (sedatives) OR (hypnotics) OR (anxiolytics) OR (tramadol))) AND DOCUMENT TYPES: (Article OR Review)  Refined by: WEB OF SCIENCE CATEGORIES: ( BEHAVIORAL SCIENCES OR PUBLIC ENVIRONMENTAL OCCUPATIONAL HEALTH OR SUBSTANCE ABUSE OR PSYCHIATRY OR PSYCHOLOGY CLINICAL OR PSYCHOLOGY MULTIDISCIPLINARY OR PSYCHOLOGY ) AND [excluding] WEB OF SCIENCE CATEGORIES: ( ENGINEERING ENVIRONMENTAL OR ERGONOMICS OR ENGINEERING INDUSTRIAL OR ENVIRONMENTAL SCIENCES OR CONSTRUCTION BUILDING TECHNOLOGY OR WATER RESOURCES OR AGRICULTURE DAIRY ANIMAL SCIENCE )  Indexes=SCI-EXPANDED, SSCI, A&HCI, CPCI-S, CPCI-SSH, BKCI-S, BKCI-SSH, CCR-EXPANDED, IC Timespan=1900-2013 |
| # 4 | [**413**](http://apps.webofknowledge.com/summary.do?product=WOS&doc=1&qid=138&SID=3D4lpT9QyE9RjUNwMDP&search_mode=CombineSearches) | #5 OR #2 OR #1  Indexes=SCI-EXPANDED, SSCI, A&HCI, CPCI-S, CPCI-SSH, BKCI-S, BKCI-SSH, CCR-EXPANDED, IC Timespan=1900-2013 |
| # 5 | [**138,495**](http://apps.webofknowledge.com/summary.do?product=WOS&doc=1&qid=140&SID=3D4lpT9QyE9RjUNwMDP&search_mode=AdvancedSearch) | TI=((tobac*) OR (smok*) OR (snuff) OR (cigarette) OR (smoker) OR (nicotine) OR (hookah) OR (narghile) OR (argila) OR (shisha) OR (waterpipe))  Indexes=SCI-EXPANDED, SSCI, A&HCI, CPCI-S, CPCI-SSH, BKCI-S, BKCI-SSH, CCR-EXPANDED, IC Timespan=1900-2013 |
| # 6 | [**175**](http://apps.webofknowledge.com/summary.do?product=WOS&doc=1&qid=142&SID=3D4lpT9QyE9RjUNwMDP&search_mode=CombineSearches) | #4 AND #5  Indexes=SCI-EXPANDED, SSCI, A&HCI, CPCI-S, CPCI-SSH, BKCI-S, BKCI-SSH, CCR-EXPANDED, IC Timespan=1900-2013 |
| # 7 | [**314,423**](http://apps.webofknowledge.com/summary.do?product=WOS&doc=1&qid=143&SID=3D4lpT9QyE9RjUNwMDP&search_mode=AdvancedSearch) | TI=((alcoh*) OR (ethanol*) OR (wine) OR (beer) OR (drink) OR (liquor))  Indexes=SCI-EXPANDED, SSCI, A&HCI, CPCI-S, CPCI-SSH, BKCI-S, BKCI-SSH, CCR-EXPANDED, IC Timespan=1900-2013 |
| # 8 | [**69**](http://apps.webofknowledge.com/summary.do?product=WOS&doc=1&qid=144&SID=3D4lpT9QyE9RjUNwMDP&search_mode=CombineSearches) | #4 AND #7  Indexes=SCI-EXPANDED, SSCI, A&HCI, CPCI-S, CPCI-SSH, BKCI-S, BKCI-SSH, CCR-EXPANDED, IC Timespan=1900-2013 |
| # 9 | [**376,891**](http://apps.webofknowledge.com/summary.do?product=WOS&doc=1&qid=166&SID=3D4lpT9QyE9RjUNwMDP&search_mode=AdvancedSearch) | TI=((marijuana) OR (THC) OR (ecstasy) OR (MDMA) OR (LSD) OR (PCP) OR (amphet*) OR (cocaine) OR (opioid*) OR (opiate) OR (narco*) OR (khat) OR (Qat) OR (crack) OR (heroin) OR (barbit*) OR (mescaline) OR (benzodiazepines) OR (ketamine) OR (diazepam) OR (codeine) OR (methylphenidate) OR (psychoactive*) OR (cannabis) OR (inhalants) OR (hallucinogens) OR (sedatives) OR (tranqu*) OR (GHB) OR (methylphenidate) OR ("street drugs") OR (methadone) OR (Buprenorphine) OR (lorazepam) OR (clonazepam) OR (methampheta*) OR (dextromethorphan) OR (cannabinol) OR (steroid*) OR (tramadol) OR (hashish) OR (volatile) OR (morphine) OR (psychotrop*))  Indexes=SCI-EXPANDED, SSCI, A&HCI, CPCI-S, CPCI-SSH, BKCI-S, BKCI-SSH, CCR-EXPANDED, IC Timespan=1900-2013 |
| # 10 | [**76**](http://apps.webofknowledge.com/summary.do?product=WOS&doc=1&qid=167&SID=3D4lpT9QyE9RjUNwMDP&search_mode=CombineSearches) | #9 AND #4  Indexes=SCI-EXPANDED, SSCI, A&HCI, CPCI-S, CPCI-SSH, BKCI-S, BKCI-SSH, CCR-EXPANDED, IC Timespan=1900-2013 |
| # 12 | [**107**](http://apps.webofknowledge.com/summary.do?product=WOS&doc=1&qid=169&SID=3D4lpT9QyE9RjUNwMDP&search_mode=AdvancedSearch) | #6 OR #8 OR #10 AND NOT #14  Indexes=SCI-EXPANDED, SSCI, A&HCI, CPCI-S, CPCI-SSH, BKCI-S, BKCI-SSH, CCR-EXPANDED, IC Timespan=1900-2013 |
